# Wing vein abnormality analysis in honeybee (*Apis mellifera* L. 1758) populations from Iran

**DOI:** 10.1038/s41598-024-70147-6

**Published:** 2024-08-20

**Authors:** Mahdi Shahshahani, Roohollah Abbasi

**Affiliations:** Department of Plant and Animal Biology, Faculty of Biological Science and Technology, University of Isfahan, Isfahan, Iran

**Keywords:** Wing vein abnormality, Forewing, Hindwing, Honeybee, *Apis mellifera*, Zoology, Entomology

## Abstract

The insect wing is one of the most important characteristics that allowed insects to occupy most of the habitats on the planet. Honeybee wings has been the subject of studies on the venation abnormalities. A total of 424 honeybees from 14 locations were collected and all four wings were removed and examined for 19 abnormalities on the forewings and 6 abnormalities on the hindwings. In general, supernumerary veins were the most common abnormalities seen and abnormalities no. 23, 2, 6, 1, 5, 21, 10, 13 had the highest and abnormalities no. 11, 17, 18, 19, 20, and 25 had the lowest frequencies. All of the abnormalities had similar frequencies in the right and left wings in the population. In terms of correlation between 25 abnormalities, abnormality pairs AB3–AB13, AB6–AB7, AB7–AB8, AB10–AB12, AB16–AB17 on the forewing and AB2–AB23, AB12–AB20, AB12–AB24, AB13–AB21, AB16–AB25, and AB17–AB25 between the forewing and hindwing show significant positive correlations and abnormality pairs AB4–AB5, AB7–AB15 and AB8–AB9 on the forewing show significant negative correlations with each other. In terms of the differential occurrence of abnormalities , a few locations differed significantly from other locations. This study provides some insights into the nature of these abnormalities on the honeybee wings.

## Introduction

One of the great resources for studying morphological diversity is the insect wing^1^. Insect wings are found on the second (forewing) and third (hindwing) thoracic segments of some insects and normally are adult structures made from the cuticles of the exoskeleton^2^. By definition, forewing and hindwing are serially homologous structures^3^. Anatomically they are made of two cellular membranes supported by tubular sclerotized structures called veins. There are two types of veins including longitudinal veins (contain blood vessels, trachea, and nerve fibers) and crossveins (transverse struts that play a structural role and make characteristic venation patterns) that form a regular pattern known as venation. Consistent wing venation patterns within groups i.e. orders and families provide features for insect identification and classification. Wing venation may become simpler by fusion or loss of veins or become more complex by the addition of numerous crossveins or substantial terminal branching^2^. Wing veins are determined during larval period but differentiates in pupal stage^4^. Based on the knowledge on *Drosophila melanogaster* wing vein development, a critical step is the activation of the decapentaplegic (Dpp)/bone morphogenetic protein (BMP) signaling pathway during pupal stage. Also, redistribution of Dpp reflecting the vein patterns, by directional transport from the longitudinal veins into the posterior crossvein by BMP-binding proteins is a key mechanism (Reviewed in Shimmi et al.^1^).

Deviations from the normal wing venation pattern in insects are called wing vein abnormalities. These abnormalities can arise from various factors and can affect the insect’s flight performance, survival, and fitness. Vein fusion, duplication, and loss are examples of abnormalities^4^. The vein fusion is caused when two or more veins merge together, resulting in a thicker vein, which can increase the risk of fracture by reducing the flexibility and resilience of the wing. The vein duplication is caused when a vein or a part of a vein is duplicated, resulting in an extra vein or a branch, which can affect the wing’s aerodynamic shape by increasing the complexity and weight of the wing. The vein loss is caused when a vein or a part of a vein is missing, resulting in a gap or a discontinuity in the wing which can impair the wing’s ability to generate lift by reducing the stiffness and strength of the wing^5–7^.

Genetic, developmental, and environmental factors may contribute to abnormalities and defects in the wing veins^4,8–11^. In a study on 29 mutations out of many known to affect wing venation patterns in *Drosophila melanogaster*, it has been demonstrated that “several genetic operations” are at work to make correct wing venation patterns^4^. Developmental buffering mechanisms make phenotypic characters stable against perturbations, but sometimes these mechanisms can be overcome by adaptive evolution. It has been demonstrated that in highland Ethiopian inbred *Drosophila melanogaster* strains that evolve larger wings, susceptibility to wing abnormalities is increased^9^. Also, it has been demonstrated that exposure to heavy metals like cadmium can cause wing abnormalities and these abnormalities can be seen in at least the next four generations through epigenetic mechanisms even in the absence of the heavy metal^8^. It was also shown that exposure to neonicotinoid insecticides increased wing venation abnormalities in honeybees^11^.

The honeybee is one of the most economically important insects due to its contribution to pollination and the products it makes^12^. There are number of reports of abnormality in the wing venation of honeybee populations from Denmark, Norway, Sweden, Austria, Croatia and Slovenia^13^, Poland^14,15^, Italy^16^, Turkey^17^, Egypt^18^, Japan^19^ and China^20^. Some studies emphasize on the role of environmental factors and other studies emphasize on the role of genetics on the wing abnormalities in honeybee. Here we examined the abnormalities on the forewing and hindwing at both left- and right-hand side of honeybees collected from different locations in central Iran. We report these abnormalities for the first time from Iran and three abnormalities on the forewing (17, 18 and 19) and two abnormalities (20 and 24) on the hindwing and correlations between all 25 abnormalities were examined for the first time.

## Results

### Number and frequency of abnormalities observed

**Figure 1:**
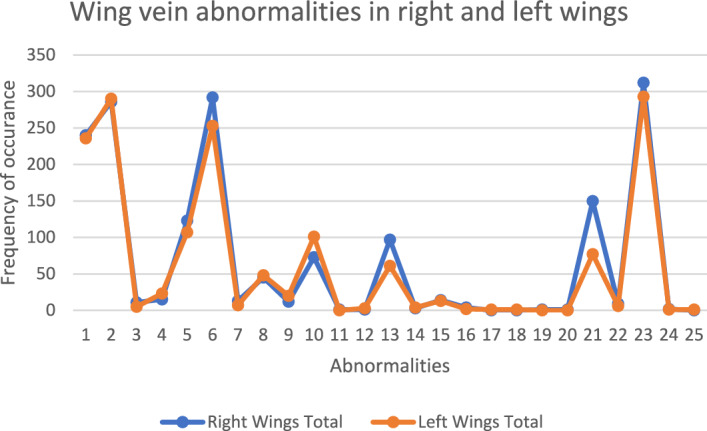
Frequency of 25 abnormalities found in the right and left forewing and hindwing of 424 honeybees. The X axis is the 25 abnormalities and the Y axis is the total number of abnormalities observed. The pattern of the graph depicts the symmetrical occurrence of abnormalities in the populations.

A total of 25 abnormalities recognized on the fore and hind wing venations based on the previous publications as well as our own examination. Each abnormality examined on right and left wings considering its quality of appearance (clear/vague) and its presence/absence (1/0) recorded in a data matrix but only total values are used in subsequent analyses. The frequency of these abnormalities is presented in Table 1 and Fig. 1. Looking at the frequency data it is clear that some of the abnormalities are more common than the others. As examples abnormalities no. 23, 2, 6, 1, 5, 21, 10, 13 have the highest frequency and abnormalities no. 11, 17, 18, 19, 20 and 25 have the lowest frequency of occurrence respectively. Also, the right and left wings are equivalent in terms of occurrence of abnormalities (Table 1 and Fig. 1).

**Table 1:** Detailed frequency of 25 abnormalities found in the right and left forewing and hindwing of 424 honeybees examined. Vague abnormality is defined as new vein formation or protrusion that is not fully completed and the vein has just become a little pointy in the spot. The percentage shows the amount of abnormality from the total observed on forewing or hindwing, i.e. abnormality 1 accounts for 19.49% of abnormalities seen on the right forewing. Only total values are used in subsequent analyses.

	Abnormality name	Abnormality frequency							
		Right				Left			
		Clear	Vague	Total	Percentage	Clear	Vague	Total	Percentage
Forewing	1	104	136	240	19.49	120	116	236	20.08
	2	80	206	286	23.23	69	221	290	24.68
	3	2	9	11	0.89	0	5	5	0.42
	4	2	13	15	1.22	9	14	23	1.96
	5	14	109	123	9.99	16	91	107	9.11
	6	38	254	292	23.72	25	228	253	21.53
	7	9	4	13	1.05	5	2	7	0.59
	8	10	35	45	3.65	16	32	48	4.08
	9	4	8	12	0.97	7	13	20	1.70
	10	9	64	73	5.93	9	92	101	8.59
	11	1	0	1	0.08	0	0	0	0
	12	1	0	1	0.08	3	0	3	0.25
	13	2	95	97	7.88	0	61	61	5.19
	14	2	1	3	0.24	4	0	4	0.34
	15	1	13	14	1.14	3	10	13	1.10
	16	4	0	4	0.32	1	1	2	0.17
	17	0	0	0	0	1	0	1	0.08
	18	0	0	0	0	1	0	1	0.08
	19	1	0	1	0.08	0	0	0	0
Hindwing	20	1	0	1	0.21	0	0	0	0
	21	10	140	150	31.64	3	74	77	20.37
	22	1	8	9	1.90	1	5	6	1.59
	23	117	195	312	65.82	108	185	293	77.51
	24	2	0	2	0.42	0	1	1	0.26
	25	0	0	0	0	1	0	1	0.26

### Symmetrical occurrence of the abnormalities

In order to further investigate the frequency of abnormalities on left and right wings in the populations, the data first checked against the normal distribution using Shapiro–Wilk’s test. All of the groups showed distributions significantly (p < 0.05) different from normal distribution as follow: right forewing (Shapiro–Wilk W = 0.6812, p = 3.346E−05), left forewing (Shapiro–Wilk W = 0.6904, p = 4.279E−05), right hindwing (Shapiro–Wilk W = 0.7162, p = 0.009114), left hindwing (Shapiro–Wilk W = 0.6495, p = 0.001739). The Kruskal–Wallis H test for non-parametric data followed by Dunn’s post hoc test was carried out to compare abnormalities between right and left wings. The result demonstrated that there were no significant differences (p < 0.05) between the abnormalities in the right and left forewings (H = 0 and p = 1) and the right and left hindwings (H = 0.1603 and p = 0.6863). Dunn’s post hoc test with Bonferroni corrected p values also showed that there were no significant differences (p < 0.05) between the occurrence of abnormalities in the right and left forewings (Z = 0 and p = 1) and the right and left hindwings (Z = 0.4039 and p = 0.6863).

### Correlation between the 25 abnormalities

**Figure 2:**
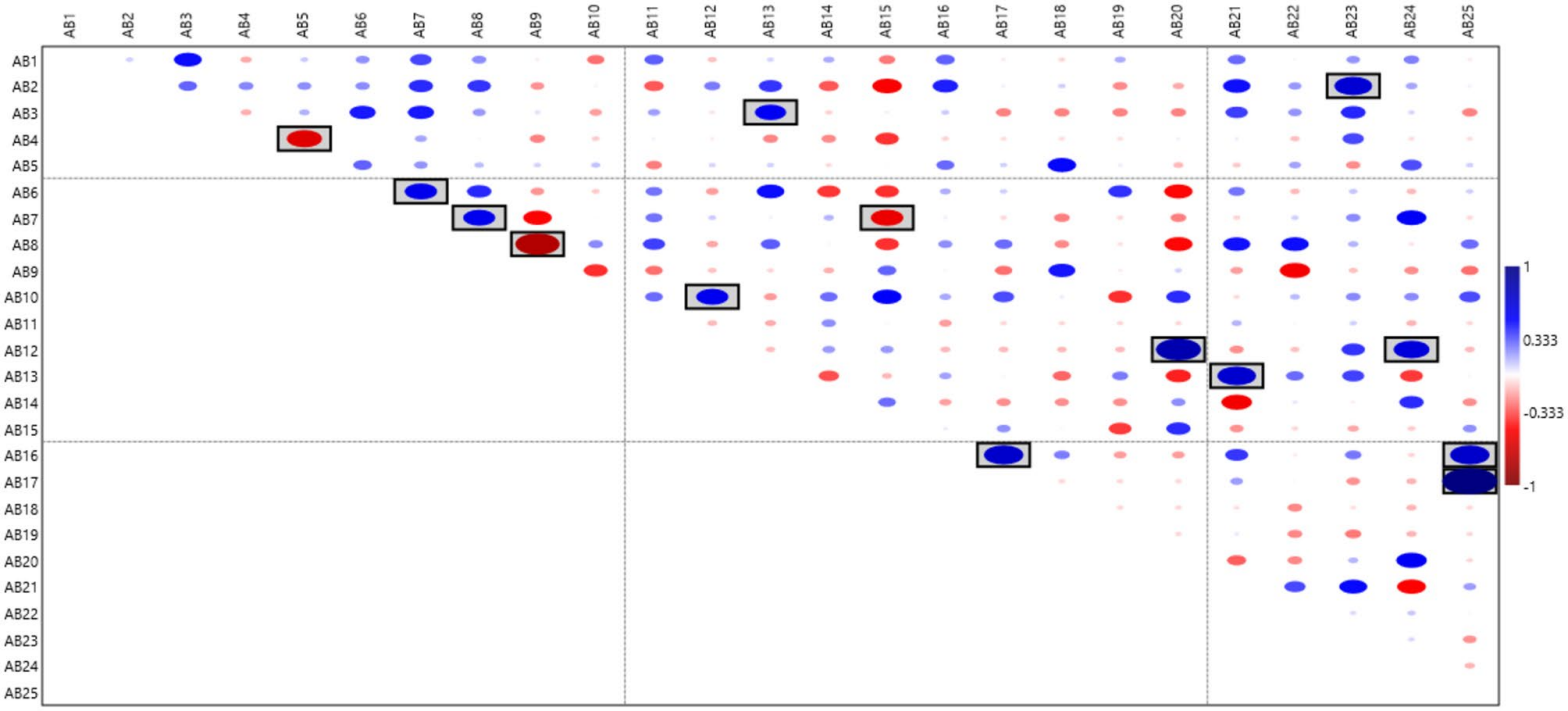
Correlation coefficient analysis of the 25 abnormalities of the forewing (AB1–AB19) and hindwings (AB20–AB25). Correlation coefficients of greater than, less than, and equal to zero indicate positive, negative, and no relationship between the variables respectively. The diameter of the ellipse shows the strength of correlation. Blue ellipse shows positive correlations and red ellipse shows negative correlations. The significant correlations (p < 0.05) indicated with boxes.

A correlation coefficient test was carried out to see if abnormalities correlated with each other in any positive or negative way. The results showed only 14 significant correlations out of 300 pairwise comparisons. Abnormality pairs AB3–AB13, AB6–AB7, AB7–AB8, AB10–AB12, AB16–AB17 on the forewing and AB2–AB23, AB12–AB20, AB12–AB24, AB13–AB21, AB16–AB25, and AB17–AB25 between the forewing and hindwing were significantly positively correlated (Fig. 2). The results also showed that abnormality pairs AB4–AB5, AB7–AB15 and AB8–AB9 on the forewing were significantly negatively correlated (Fig. 2). However, taking Bonferroni correction into account left only the correlation between AB17 (on the forewing) and AB25 (on the hindwing) as significant which seems to be type II error (false negative) due to high number of pairwise comparisons.

### Geographic locations and the abnormalities

In order to investigate whether the occurrence of the abnormalities is different in different sampling locations, the data were first checked against the normal distribution using a Shapiro–Wilk’s test. The data for location 4 showed a non-normal distribution (W = 0.9267, p = 0.04025) therefore we decided to do a non-parametric analysis of variance. A Kruskal–Wallis H test for non-parametric data was used to compare 14 populations from different locations. The results of Kruskal–Wallis H test demonstrated a significant difference for occurrence of abnormalities in different locations, H (chi2) = 50.09, Hc (tie corrected) = 50.8, p = 0.000002176. In general, Dunn's post hoc test for pairwise comparison with Bonferroni corrected p values demonstrated that out of 91 pairwise comparisons, most of the locations were similar in terms of occurrence of abnormalities. The results also showed that occurrence of abnormalities in Khatoonabad (location 12) and Farahan (location 14) differ significantly from a few other locations (Table 2).

**Table 2:** Dunn’s post hoc test for pairwise comparison of 14 populations in terms of 25 abnormalities. Location number is based on Table 3. Numbers on the right are Bonferroni corrected p values and numbers on the left are Z statistics. P values lower that 0.05 are considered statistically significant and are shown in bold font. Uncorrected p values are provided in supplemental data file.

Location	1	2	3	4	5	6	7	8	9	10	11	12	13	14
1		1	1	1	1	1	1	1	1	1	1	**0.006495**	1	**0.01375**
2	1.052		1	1	1	1	1	1	1	1	1	0.3188	1	0.5638
3	0.4318	0.6201		1	1	1	1	1	1	1	1	**0.03643**	1	0.07161
4	1.633	0.5814	1.202		1	1	1	0.6379	1	1	1	1	1	1
5	0.2255	0.8265	0.2064	1.408		1	1	1	1	1	1	**0.01634**	1	**0.03327**
6	1.229	0.1772	0.7973	0.4042	1.004		1	1	1	1	1	0.5548	1	0.9523
7	1.728	0.6764	1.296	0.09495	1.503	0.4992		0.4777	1	1	1	1	1	1
8	1.063	2.115	1.495	2.696	1.289	2.292	2.791		1	1	1	**4.36E-05**	1	**0.0001111**
9	0.3803	0.6716	0.05146	1.253	0.1549	0.8488	1.348	1.443		1	1	**0.02994**	1	0.05937
10	0.6429	0.409	0.2111	0.9904	0.4175	0.5862	1.085	1.706	0.2626		1	0.07939	1	0.1505
11	0.8224	0.2619	0.3773	0.8612	0.59	0.4445	0.9591	1.918	0.4303	0.1597		0.09735	1	0.1862
12	3.972	2.92	3.54	2.338	3.746	2.743	2.243	5.035	3.591	3.329	3.271		**0.00525**	1
13	0.05039	1.102	0.4822	1.684	0.2758	1.28	1.779	1.013	0.4307	0.6933	0.8743	4.022		**0.01122**
14	3.789	2.737	3.357	2.156	3.564	2.56	2.061	4.852	3.409	3.146	3.083	0.1825	3.84	

## Discussion

Extra or missing veins, incomplete veins and vein protrusions caused the wing venation pattern of some individuals slightly defer from the population. Here we examined a total of 25 abnormalities on all 4 wings of honeybee. The abnormalities were defined based on previous publications^13–19^ except for abnormalities 17, 18 and 19 on the forewing and the abnormalities 20 and 24 on the hindwing which were defined and examined for the first time. Based on the frequency of the abnormalities recorded, it appears that the abnormalities no. 23 (M), 2 (Rs), 6 (1m-cu), 1 (2rs-m), 5 (Rs 2nd abscissa), 21 (M), 10 (1rs-m and M), 13 (2m-cu) had the highest frequency and abnormalities no. 11 (1rs-m and M), 17 (Rs), 18 (Rs), 19 (M + Rs and Cu), 20 (M and Cu) and 25 (M) had the lowest frequency of occurrence in the population respectively. Supernumerary veins are the most common abnormalities seen in our study which is consistent with previous findings in honeybees^16–18^ and solitary wasps of families Sphecidae^21^ and Crabronidae^22^. Occurrence of abnormalities with different frequencies has been reported in honeybees before. Maa and Shae reported abnormalities AB2 (on Rs) and AB1 (on 2rs-m) and position 1rs-m as the most, and an abnormality on 2m-cu as the least frequent abnormalities and variable positions in *Apis cerana* Fabricius worker population from South China^19^. According to Adolph (1880, cited in^23^ and described in^19^), occurrence of abnormality AB1 seems to be a common phenomenon in bees and other Aculeata. In general, crossvein 1rs-m, 1m-cu and 2m-cu also reported to be most variable positions^19,20,23^. Comparing Carniolan (*A.m. carnica*) and Egyptian (*A.m. lamarckii*) bee populations, Mazeed^18^ found that the former shows higher rate of vein abnormality than the later. He also found that in the forewing, marginal cell and first cubital cell have the highest and lowest abnormal extra veins respectively and extra veins (spurs) are more common than incomplete veins. On the hindwing, extension of medial vein was only abnormality found^18^. Porporato et al. (2014) demonstrated that on the right forewing of Italian honeybee populations, in general spurs with various length are the most frequent abnormalities and among them, abnormalities equivalent to AB1, AB5 (on Rs) and AB2 are rather common. Łopuch and Tofilski studied wing vein abnormalities on the forewings in workers, drones and queens of honeybee populations from Poland and among 15 veins and crossveins with abnormalities, reported that abnormalities on 2rs-m, M, 1rs-m, 1m-cu, Cu and Cu2 are common in all castes. Eligül et al. (2017) studied left forewings of 23 honeybee populations throughout Turkey and reported that AB2 and AB1 was the most common and AB11 (on 1rs-m) was the least common abnormalities observed. All of these suggest that some parts of the wing probably are more susceptible to the abnormalities in vein formation than the others.

Frequencies of wing vein abnormalities on the left and right wings in the populations were investigated. Even though we found differences in the frequency of each of 25 abnormalities on the wings, the frequency of the same abnormality on the left and right wings in the population was similar. However, this does not necessarily mean that in a particular sample each of these abnormalities occurred symmetrically. Overall, this finding suggests that there are common mechanisms responsible for these abnormalities in the right and left forewings and in the right and left hindwings^19^. Insects are in Bilateria lineage of the animal kingdom and these animals generally have bilateral symmetry meaning that the two sides of their body should be mirror image of each other. However, minor violations to this general rule exist in the form of many directional asymmetries, fluctuating asymmetries and antisymmetries^24^. Wings in honeybee have shown to have both symmetrical^25^ and asymmetrical morphology^14^.

Based on Cheverud’s conjecture, using phenotypic correlations as proxies for genetic correlation is justified in evolutionary biology^26^. Therefore, we used correlation analysis to get some insights into the possible underlying genetic relationships between abnormalities. For the first time, correlations between 25 wing vein abnormalities were investigated. Out of 300 pairwise comparisons, only 14 significant correlations were observed. Abnormality pairs AB3–AB13, AB6–AB7, AB7–AB8, AB10–AB12, AB16–AB17 on the forewing and AB2–AB23, AB12–AB20, AB12–AB24, AB13–AB21, AB16–AB25, and AB17–AB25 between the forewing and hindwing were positively correlated. The results also showed that abnormality pairs AB4–AB5, AB7–AB15 and AB8–AB9 on the forewing were negatively correlated. Positive correlations between abnormality pairs AB3–AB13, AB6–AB7 and AB16–AB17 probably reflect the similar genes and processes that are involved in the development of those veins. AB7 and AB12 seem to be incomplete manifestations of AB8 and AB10, respectively, so they can be considered to be synonymous. The positive correlations between abnormality pairs from forewing and hindwing (AB2–AB23, AB12–AB20, AB12–AB24, AB13–AB21, AB16–AB25, and AB17–AB25) probably reflect the similar genes and processes that govern the development of the two wings since the forewing and hindwing are serially homologous structures. AB4 and AB5 occur as protrusions in the opposite directions in the middle part of crossvein Rs-2nd abscissa, therefore it is not strange to see these two abnormalities correlate negatively. AB7 and AB15 both are protrusions from medial vein towards the crossvein 1rs-m. Negative correlations between these two abnormalities suggests that AB15 probably is an extension of AB7. The AB8 and AB9 are both protrusions from medial vein towards the crossvein 1rs-m. Negative correlations between these two abnormalities suggest that these two probably are the same and should not be separated. Overall, it seems that most of these 25 abnormalities occur independently of one another.

Geographic sampling locations in the study differ in terms of elevation, vegetation, precipitation, temperature, pollution and so on. We investigated if the occurrence of the wing vein abnormalities is different between these locations. The data suggests that occurrence of abnormalities differ significantly between a few different locations and environments. Study on the left forewing abnormalities in worker honeybees from Turkey^17^ also showed significant differences between locations. They found that two locations in southern part of the country show significantly higher percentage of abnormalities than central and northern part of the country^17^. Higher frequency of particular abnormalities in different locations was also shown in honeybees^19^. This differential variation is also seen in studies on solitary wasps^21^ and fruit flies^9^. Overall, these data suggests that environmental conditions contribute to the occurrence and frequency of these abnormalities.

## Conclusion

Authors studying wing vein abnormalities in honeybees have proposed a number of hypotheses to explain their observations i.e. effect of genetic factors^19,23,27,28^, non-genetic/environmental factors^29^ and interplay between them^19^. From the genetic factors point of view, it is demonstrated that drones (haploid males) show more abnormalities on the wing venation pattern than workers (diploid female)^13,14,19,23,28^. It is also shown that in hybrid bee strain “Nigra” which is a hybrid of European dark bee (*Apis m. mellifera*) and Carniolan honeybee (*Apis m. carnica*), female hybrid bees have higher rate of abnormalities than the females of the two original subspecies^13^. Our analysis of the abnormalities suggested that some of the abnormalities have higher frequencies than the others, some of the abnormalities  positively or negatively correlate with each other, and most of the abnormalities happen independently. These findings indirectly suggest the role of genetic factors in the phenomenon. From the non-genetic/environmental factors point of view, studies showed that honeybee wings from particular geographic locations^17,30^ and rearing condition and temperature^14,16,31,32^ differ significantly in terms of occurrence of abnormalities. Our data also confirmed that honeybee populations from different locations can show different frequency of abnormalities that emphasizes on the possible effect of environment on occurrence of abnormalities. Studies suggest that the honeybee population ontains low genetic diversity that makes it vulnerable to disease, parasites, and other environmental stressors^33,34^. Taking evolutionary perspectives into account, among winged insect lineages, Hymenoptera have relatively simple wing venation pattern and within Hymenoptera, Apocrita group including parasitoid wasps, wasps, bees, ants etc. have simpler wing venation pattern^35^. This means that in the course of evolution; honeybees have lost some of their wing veins and crossveins. Some authors have hypothesized that at least number of these abnormally appearing veinlets are probably reacquisition of ancient wing veins and crossveins that have been lost during honeybee wing evolution^14,19,30^. Some of the correlated pairs of abnormalities in this study support this hypothesis. Overall, this study in agreement with previous studies provide some clues to the reasons behind wing vein abnormalities.

## Materials and methods

### Sampling, sample preparation and imaging

Worker bees were collected from 14 apiaries located in Isfahan, Chaharmahal and Bakhtiari and Markazi provinces in central Iran (Table 3) during spring and summer of 2022 and 2023 and were transferred to the laboratory and placed in ethanol. The slide preparation and imaging were carried out based on previous publications^36,37^. Briefly, the right and left forewings and hindwings of 424 bees were carefully removed and placed on the slide. Damaged wings were not used in making slides. Wing slides were prepared by adding a drop of ethanol to the wings and putting coverslips on the wings. An image analysis system composed of a Nikon SMZ-2B stereomicroscope (Nikon: 9–16, Ohi 3-Chome, Shinagawa-Ku, Tokyo 140, Japan) and a digital microscope camera from Celestron (Celestron: 2835 Columbia St. Torrance, California 90503, USA) connected to a computer was used to image prepared slides immediately. The slides were placed in the same and similar direction under the stereomicroscope as much as possible, and all the images were taken with a magnification of 8×.

**Table 3:** The sampling locations, the total number of samples collected and the number of slides made are shown. The location number are used in subsequent analyses.

Location no	Province	Sampling location	Geographical coordinates	Elevation	Number of prepared slides
1	Isfahan	Daran (Ashan)	33°05′40.5″ N 50°40ʹ28.1″ E	2200	30
2	Markazi	Farahan	34°29ʹ48.5″ N 49°40ʹ47.4″ E	1779	30
3	Markazi	Shazand	33°56ʹ43.2″ N 49°23ʹ08.2″ E	1914	30
4	Chaharmahal and Bakhtiari	Ghale salim	32°05ʹ25.8″ N 50°56ʹ46.7″ E	2034	30
5	Chaharmahal and Bakhtiari	Farsan	32°14ʹ47.2″ N 50°33ʹ26.7″ E	2072	30
6	Isfahan	Najafabad (Yazdanshahr)	32°37ʹ06.9″ N 51°19ʹ48.4″ E	1600	30
7	Markazi	Khomeyn	33°37ʹ18.6″ N 50°03ʹ24.8″ E	1779	30
8	Isfahan	Khansar	33°11ʹ39.6″ N 50°22ʹ09.3″ E	2215	30
9	Isfahan	Golpayegan	33°29ʹ03.5″ N 50°22ʹ22.4″ E	1830	30
10	Chaharmahal and Bakhtiari	Shahrekord	32°17ʹ47.1″ N 50°52ʹ53.4″ E	2070	30
11	Isfahan	Jozdan	32°33ʹ27.0″ N 51°22ʹ42.4″ E	1600	34
12	Isfahan	Khatoonabad	32°39ʹ41.0″ N 51°47ʹ21.0″ E	1575	30
13	Isfahan	Gavart	39°39ʹ09.2″ N 51°48ʹ57.8″ E	1575	30
14	Markazi	Farahan	34°29ʹ48.5″ N 49°40ʹ47.4″ E	1779	30

### Scoring the wing vein abnormalities

**Figure 3:**
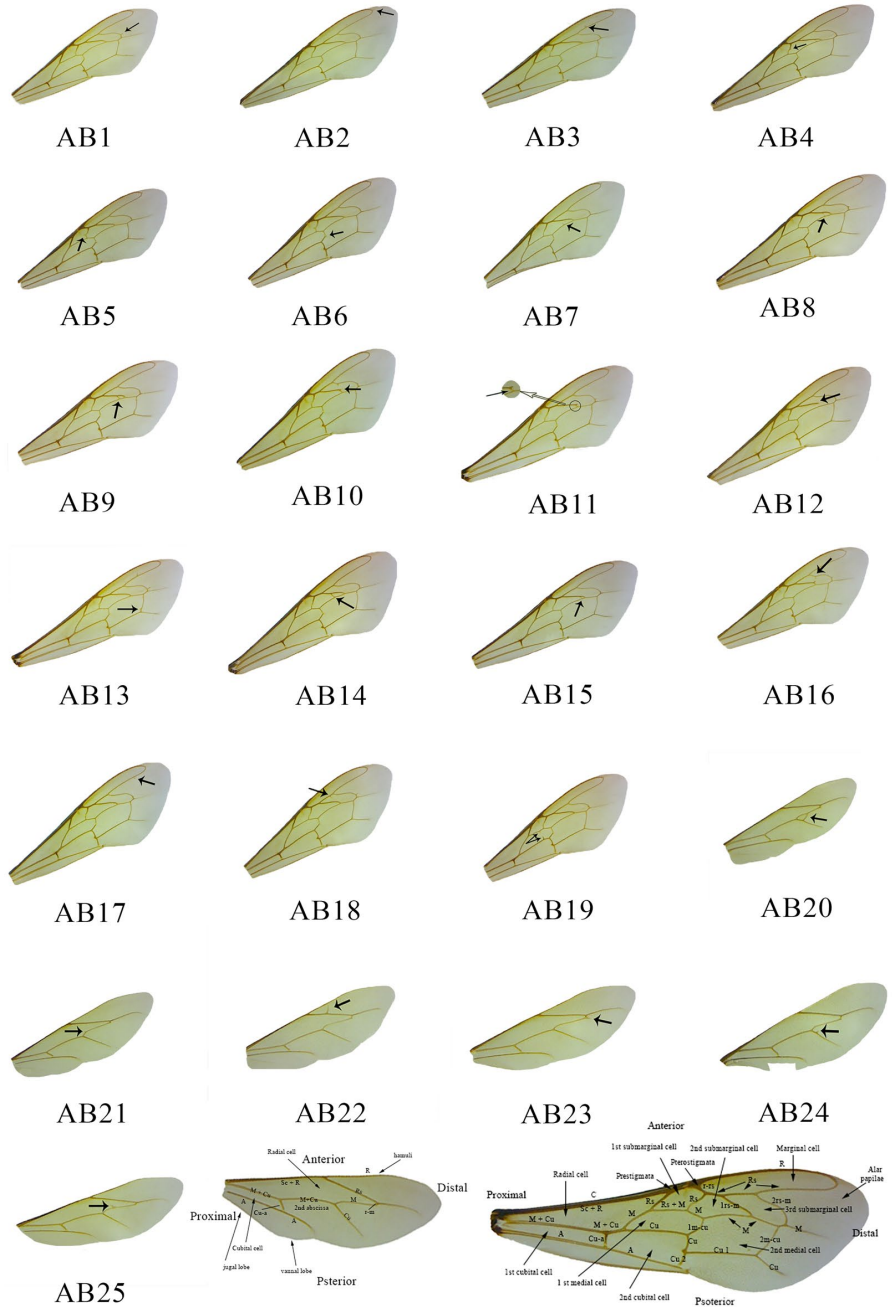
Wing vein abnormalities in the forewing and hindwing of the honeybee. Abnormalities 17, 18 and 19 on the forewing and the abnormalities 20 and 24 on the hindwing were examined for the first time. Terminology of the veins is based on Engel^39^. *A* anal vein, *C* costal vein, *Cu* cubital vein, *M* medial (basal) vein, *R* radial vein, *Rs* radial sector, *Sc* subcostal vein. Abnormalities 1–25 are defined as follows: AB1: Seemingly new vein formation from 2rs-m crossvein towards proximal part of the wing, protruding parts differ in length among samples. AB2: Protruding part is at the end of the R vein and differs in length among samples. AB3: In the middle parts of Rs vein, a new vein protrusion was seen towards anterior part of the wing. AB4: Vein protrusion seen near middle part of the Rs (2nd abscissa) vein, pointing towards proximal part of the wing. AB5: Near the middle of Rs (2nd abscissa) vein, and in about the same spot as abnormality number 4, but pointing towards distal part of the wing. AB6: On 1 m-cu crossvein, protruding towards distal part of the wing. AB7: Incomplete vein formation between 1rs-m crossvein and M vein. AB8: Vein formation between 1rs-m crossvein and M vein is incomplete, unlike abnormality number 7, vein protrusion is seen at both ends. AB9: New vein is formed, connecting 1rs-m crossvein and M vein making a medium size cell. AB10: 1rs-m crossvein is continuing straight instead of turning down. AB11: 1rs-m crossvein has been slightly faded in the connecting part to the M vein. AB12: Like abnormality number 10, the vein heads straight, but the connection with the M vein is lost. AB13: Small vein protrusion is seen in lower part of 2m-cu crossvein. AB14: Like abnormality number 9, connection between two veins is formed, but the connection is more to the proximal part of the wing making larger cell. AB15: Like abnormality number 9, new connection between 1rs-m crossvein and M vein is formed, but more towards the distal part of the wing making a smaller cell. AB16: The protrusions are seen at both 1rs-m and 2rs-m crossveins, reaching towards each other to divide 3rd submarginal cell. AB17: Small vein protrusion is seen on distal part of the Rs vein pointing towards posterior part of the wing. AB18: On middle Rs vein, the protrusion is seen pointing towards 1rs-m crossvein. AB19: The vein protrusions are seen on both Rs + M and Cu pointing towards each other. AB20: New vein connection is formed between M and Cu veins. AB21: Small vein formation is seen on the M vein; the new protrusion is pointing towards anterior part of the wing. AB22: On the Rs vein, the protrusion is facing towards anterior part of the wing. AB23: New vein protrusion is seen along vein M. AB24: Here the new protrusion is seen on vein Cu pointing towards vein M. Ab 25: Vein protrusion is on the middle part of the vein M, pointing towards anterior part of the wing.

Abnormalities such as incomplete veins, vein protrusions as well as extra/missing veins were defined based on the previous studies^13–19,32^ and 3 abnormalities on the forewing and 2 abnormalities on the hindwing were examined for the first time. Of the total of 25 abnormalities recognized, 19 occur in the forewing and 6 occur in the hindwing (Fig. 3). The abnormalities were divided and scored based on clarity of occurrence as well. A data matrix was prepared in which the bee individuals and populations were placed in rows and abnormalities separated by each four wings were placed on columns. The presence and absence of each abnormality were marked by 1 and 0 in the data matrix respectively. The frequency of abnormality in each individual was calculated by adding up the numbers in rows and the frequency of each abnormality was calculated by adding up the numbers in columns.

### Statistical analysis of the data

Statistical analyses were carried out using the PAST v4.12 software package^38^. In order to examine abnormalities in terms of symmetrical occurrence in populations, first the data were checked for normality using Shapiro–Wilk’s test. The Kruskal–Wallis H test for non-parametric data followed by Dunn’s post hoc test was used to  separately compare right and left forewing as well as right and left hindwing with each other. To investigate if the abnormalities correlated with each other in positive or negative way, a correlation coefficient analysis was carried out. After checking the data for normality of distribution using Shapiro–Wilk’s tests, a Kruskal–Wallis H test is used to compare equal medians of abnormalities among 14 populations. Then the Dunn’s post hoc test was used to compare 14 populations with one another.

### Supplementary Information

Supplementary Information.

## Data Availability

Venation abnormality data is available as an Excel file.
